# A meeting of positive behaviors: The relations of three aspects of flexibility with character strengths

**DOI:** 10.3389/fpsyg.2022.1078764

**Published:** 2023-02-03

**Authors:** Valentina Vylobkova, Sonja Heintz

**Affiliations:** ^1^Department of Psychology, University of Zurich, Zurich, Switzerland; ^2^School of Psychology, Faculty of Health, University of Plymouth, Plymouth, United Kingdom

**Keywords:** flexibility, adaptability, character strengths, CSV classification, daily diaries

## Abstract

**Introduction:**

The adaptation of own plans and behaviors to new circumstances seems to be a desirable personal quality in the modern world. it has been assumed that adaptability cannot be transferred to a single character strength.

**Methods:**

The present research examines this assumption using typical and daily behaviors of three aspects of flexibility (predictability, adaptability and orderliness) and 24 character strengths across two studies (*N_1_ =* 283, *N_2_ =* 188).

**Results:**

Flexibility showed a consistent and large overlap with character strengths. Adaptability was positively related to most of the strengths. Predictability was positively related to humility and prudence, and orderliness to perseverance, prudence, and selfregulation.

**Discussion:**

These results support our initial assumption and build a strong basis for further examining the relationships and interplay between flexibility and character strengths. They also constitute an important first step toward integrative positive interventions that target relevant aspects of flexibility and character.

## 1. Introduction

Already in 1954, Paul R. Lawrence, former Professor of Organizational Behavior at Harvard Business School, indicated that resistance to change is one of the most common and trouble-causing problems of an organization (Lawrence, 1954). In today’s unpredictable world, which was termed ‘VUCA-world’ (Volatile, Uncertain, Complex, and Ambiguous environment, Baran and Woznyj, 2021), people face changes not only at the workplace but in almost every area of everyday life. Therefore, successfully dealing with changes and the ability to adapt one’s plans, behaviors, and thoughts to new circumstances seem to be crucial in modern society. The importance of flexible and adaptive behaviors was further highlighted in the last 2 years during the global pandemic and restrictive governmental measures that required an enormous amount of flexibility from the citizens. People needed to adapt to home office and homeschooling, to video meetings and social distancing. Flexibility appears not only to be an important personal quality, but also a positive one. On a societal level, it is one of the 21st-century competences and is seen as a desirable quality to develop in adolescents, aiming to prepare them for the world they will live and work in (Ananiadou and Claro, 2009).

Flexibility and its aspects have been studied in a number of psychological subdisciplines, such as personality psychology (Bitterwolf, 1992; Hossiep and Paschen, 1998) or work and organizational psychology (Ployhart and Bliese, 2006). Flexibility is a relevant personal quality in the modern world, and—on the level of cognition—is a key component of mental health (Kashdan and Rottenberg, 2010). Further research underlines the positive role of flexibility and related concepts for well-being (e.g., for overviews on psychological flexibility see Doorley et al., 2020; Stenhoff et al., 2020). In an attempt to unify these approaches, a recent flexibility measure outlines three aspects of flexibility (Vylobkova and Heintz, 2022a): Predictability (planning daily life), adaptability (dealing well with change) and orderliness (following order and rules). Overall, a person is more flexible the less predictable and the more adaptive they are. Further, a certain degree of orderliness is needed to coordinate and implement changes. As personality is an aggregate of different traits, it is important to understand their inter-relations in order to explain human behaviors. Yet, very little is known about the relationship between flexibility and other personal traits. In the present research, we focus on flexibility as a personality dimension and adopt a recent measure of flexibility (Vylobkova and Heintz, 2022a) to study its relationships with morally valued personality aspects that describe “a good character.”

Human character is a well-studied concept in psychology, especially since the rise of positive psychology (Seligman and Csikszentmihalyi, 2000). To date, several approaches to the human character exist in the literature, with the most prominent one being the Classification of Character Strengths and Virtues (CSV) by Peterson and Seligman (2004). The CSV classification describes what is ‘right’ about people in 24 character strengths, such as love of learning, curiosity, humility, fairness or zest. Character strengths are positive personality traits that are relatively stable but malleable. They display distinct ways to achieve the six universal core virtues to which they are theoretically assigned: Wisdom and knowledge, courage, humanity, justice, temperance, and transcendence (Peterson and Seligman, 2004; see Supplementary Table S1).

Character strengths were related to a large number of desirable outcomes, such as general life satisfaction (e.g., Park et al., 2004; Heintz and Ruch, 2020), student satisfaction, positive classroom behavior, and school achievement (e.g., Lounsbury et al., 2009; Wagner and Ruch, 2015; Wagner et al., 2020), work engagement (e.g., Harzer and Ruch, 2013), and different aspects of job performance (e.g., Avey et al., 2012; Harzer and Ruch, 2014). Research has also shown the positive role of character strengths in enhancing individual well-being (e.g., Proctor et al., 2011; Gander et al., 2013; Harzer and Ruch, 2016; for an overview, see Schutte and Malouff, 2019). In the domain of interpersonal relationships and human development, character strengths are seen as positive and desirable qualities: Future parents wish certain character strengths for their children rather than intelligence (Wagner et al., 2019). Character strengths are also the qualities adolescents are looking for in a friend (Wagner et al., 2019). As the main focus of this research was put on the relevance of character strengths for well-being, and evaluating character-strengths interventions, rather little is known about the relations between the 24 character strengths and other positive aspects of human nature (for exceptions, see Martínez-Martí and Ruch, 2017; Moeller and Stahlmann, 2019). The present study seeks to help fill this gap by investigating how character strengths relate to flexibility.

We regard flexibility as a positive personality trait. In the present research, we seek to investigate relationships between two sets of positive traits, the 24 character strengths and the three flexibility dimensions, to determine to what extent these positive traits overlap. Lavy (2020) suggests that 21st-century competences are closely related to character strengths, but not every competence can be transferred to a single character strength. One of these non-transferrable competences is adaptability, which raises several questions: How is flexibility (and its aspects, including adaptability) related to character strengths? How close are those overlaps and do they differ for the three aspects of flexibility? Are those overlaps observable on the level of typical behaviors (i.e., traits) and on the level of daily behaviors (i.e., states)? This would highlight not only the positivity of flexibility, but also to what extent the flexibility dimensions tap into moral dimensions of character.

In the present research, we investigate these questions in two empirical studies. We first explore the relationships between flexibility and character strengths at the level of typical behaviors (or traits) in Study 1. In Study 2, we seek to replicate these initial results and expand them by exploring the relationships at the level of daily behaviors. The importance of studying both levels is highlighted in a number of personality theories (e.g., Fleeson, 2001; Roberts, 2018), as there can be either matches or discrepancies between states and traits.

## 2. Study 1: Exploring the relationships on the general level of typical behaviors

To our knowledge, there has been no empirical evidence on the relationships between flexibility and the 24 character strengths (Peterson and Seligman, 2004). With Study 1, we aim to initially explore these relationships at the level of typical behaviors; that is, the stable patterns that can be observed in behavior, thoughts and feeling of a person over a number of different situations (i.e., traits). We expect that flexibility and character strengths overlap as follows, based on empirical studies (e.g., Dametto and Noronha, 2021; Vylobkova and Heintz, 2022a) as well as conceptual similarities: (1) Predictability will be negatively related to character strengths in general. (2) Adaptability will be positively related to character strengths in general, especially to curiosity and creativity. (3) Orderliness will be positively related to self-regulation and perseverance.

### 2.1. Materials and methods

#### 2.1.1. Participants

We aimed to recruit adult participants who had at least 50% workload and had a good command of German. Participants were recruited via different social-media channels (e.g., Facebook, LinkedIn), advertisements in companies, and via personal contacts in Switzerland, Germany, and Austria. The necessary sample size was chosen regarding test power, requirements for a planned missing design (SAPA; Revelle et al., 2017), and considerations on the stability of correlation coefficients (Schönbrodt and Perugini, 2013). Considering these criteria, a sample size of 200 participants was determined as a minimum.

A total of 283 employees from Switzerland, Germany, and Austria completed the online survey. Four participants were excluded from the analysis due to missing data or lack of variance in the answers. The final sample consisted of 279 participants, where 25.1% were male and 74.5% were female. The age of participants ranged from 18 to 71 years (*M*_age_ = 40.86 years, *SD* = 11.92). The majority of the participants were Swiss (62.0%), followed by German (34.4%), and Austrian (3.2%). The sample was rather well educated: 60.2% of participants held a university degree, 7.8% held a PhD, and 14.7% completed an apprenticeship. Various occupational fields were listed, with the largest group from social occupations (21.9%), followed by human resources (21.5%), service sector (12.9%), scientific occupations (12.5%), and management (11.5%). Most of the participants worked full-time (*M*_wload_ = 86.1%, *SD* = 15.9%).

#### 2.1.2. Instruments

To assess the 24 character strengths, the German version of VIA-Inventory of Strengths (VIA-IS) was used (Peterson et al., 2005; German version by Ruch et al., 2010). This questionnaire consists of 240 items and assesses 24 character strengths of CSV classification, with 10 items per strength. The VIA-IS uses a 5-point Likert scale from 5 – “very much like me” to 1 – “very much unlike me.” An example item for the creativity scale is: “Being able to come up with new and different ideas is one of my strong points.” The internal consistency of the VIA-IS in the current sample was between Cronbach’s α = 0.56 (honesty) and 0.89 (spirituality), with a median of 0.76. To reduce the length of the survey with minimum impact on the reliability and validity of the measurement, a planned missing design was applied to the VIA-IS (SAPA; Revelle et al., 2017); in this design, each participant completed a random set of 96 items (4 out of 10 randomly assigned pages with 24 items each, one item per character strength) instead of all 240 VIA-IS items. The achieved reliability in this study was similar to the published German version using the full item set (Ruch et al., 2010, median 0.81), supporting the equivalence of this shortened SAPA-version with studies using all items.

To measure flexibility, the initial version of the Flexibility Scale (FS-24; Vylobkova and Heintz, 2022a) was used. The FS-24 consists of three subscales, predictability (e.g., “I feel uncomfortable with activities that are not clearly defined”), adaptability (e.g., “I can adjust my plans to changing conditions”), and orderliness (e.g., “I like to plan things in advance”), with 7–10 items per subscale. The internal consistencies of the initial FS-24 subscales in Study 1 were Cronbach’s α = 0.86 (predictability), 0.81 (adaptability), and 0.83 (orderliness).

#### 2.1.3. Procedure

The data for Study 1 was collected within a larger research project, of which parts of the data were published previously (Vylobkova and Heintz, 2022a), though none of the current research question overlap with the previous publication. Every participant provided informed consent before participation, and the study was conducted in accordance with the University’s ethics committee. All questionnaires were self-reports and were completed online. Participants first gave some demographical information, and then completed then VIA-IS, followed by the initial version of FS-24. As an incentive, participants received individual feedback on their character strengths and flexibility. Companies whose employees took part in the survey received on demand a company feedback on the company’s character strengths, accumulated over all participating employees. An additional incentive for participants was a small donation (of 1 Swiss Franc per completed survey) for a social or environmental project chosen by the participant. The donation was made by the research team for every completed survey.

#### 2.1.4. Statistical analyses

The data was analyzed with R software version 4.2.0. (R Development Core Team, 2020). We used the following R-packages: *BayesFactor* (Morey and Rouder, 2021), *dplyr* (Wickham et al., 2020), *psych* (Revelle, 2019), *tidyverse* (Wickham et al., 2019), and *lm.beta* (Behrendt, 2022). We calculated Pearson correlations to explore the relationships between the 24 character strengths and flexibility. To evaluate the results of correlational analyses, we used three criteria: Effect sizes (Gignac and Szodorai, 2016), 95% confidence intervals, and Bayes Factors (*BF*; Wetzels et al., 2011). An effect was found meaningful when all three criteria were fulfilled: At least a small effect size (*r* ≥ |0.10|), confidence interval not including zero, and *BF* ≥ 3, suggesting substantial evidence for the effect. Additionally, we computed the multiple correlation coefficient using regressions with the 24 character strengths as predictors and each of the flexibility aspects as criteria to define the overall overlap between the constructs. Effect sizes for *R^2^* were interpreted following Cohen’s (1992) guidelines, with ≥0.06 interpreted as small, ≥ 0.13 as medium, and ≥ 0.26 as large effects.

### 2.2. Results and discussion

Descriptive statistics of the study variables are presented in Supplementary Table S2. The correlations between the initial FS-24 and VIA-IS are displayed in the Table 1.

**Table 1:** Study 1: Pearson correlations between flexibility and VIA character strengths.

VIA character strengths	Flexibility subscales								
	Predictability			Adaptability			Orderliness		
	*r*	95% *CI*	*BF*	*r*	95% *CI*	*BF*	*r*	95% *CI*	*BF*
Creativity	**−0.25**	**[−0.37, −0.13]**	**>100**	**0.30**	**[0.18, 0.42]**	**>100**	**−0.18**	**[−0.30, −0.04]**	**4.04**
Curiosity	**−0.18**	**[−0.31, −0.05]**	**5.30**	**0.32**	**[0.20, 0.44]**	**>100**	−0.06	[−0.19, 0.08]	0.22
Judgment	−0.03	[−0.16, 0.10]	0.17	**0.22**	**[0.09, 0.34]**	**23.40**	0.15	[0.02, 0.28]	1.60
Love of learning	**−0.20**	**[−0.33, −0.07]**	**12.29**	**0.21**	**[0.08, 0.33]**	**16.22**	−0.10	[−0.23, 0.03]	0.49
Perspective	−0.11	[−0.24, 0.03]	0.53	**0.25**	**[0.13, 0.37]**	**>100**	0.05	[−0.08, 0.19]	0.22
Bravery	**−0.22**	**[−0.35, −0.09]**	**35.13**	**0.33**	**[0.20, 0.44]**	**>100**	0.13	[0.00, 0.26]	1.05
Perseverance	0.02	[−0.11, 0.15]	0.16	0.14	[0.01, 0.27]	1.19	**0.31**	**[0.18, 0.42]**	**>100**
Honesty	0.00	[−0.14, 0.13]	0.16	**0.18**	**[0.05, 0.31]**	**5.04**	**0.19**	**[0.06, 0.32]**	**7.47**
Zest	**−0.24**	**[−0.36, −0.11]**	**63.50**	**0.24**	**[0.11, 0.36]**	**73.95**	**0.20**	**[0.07, 0.32]**	**10.39**
Love	**−0.24**	**[−0.36, −0.11]**	**64.60**	**0.20**	**[0.06, 0.32]**	**9.63**	−0.11	[−0.24, 0.03]	0.51
Kindness	**−0.25**	**[−0.37, −0.12]**	**>100**	**0.27**	**[0.14, 0.39]**	**>100**	−0.02	[−0.15, 0.12]	0.16
Social intelligence	**−0.17**	**[−0.30, −0.04]**	**3.77**	**0.31**	**[0.18, 0.42]**	**>100**	−0.04	[−0.18, 0.09]	0.19
Teamwork	**−0.19**	**[−0.31, −0.06]**	**6.98**	**0.23**	**[0.10, 0.35]**	**42.34**	−0.03	[−0.16, 0.11]	0.17
Fairness	−0.07	[−0.21, 0.06]	0.28	**0.17**	**[0.04, 0.29]**	**3.15**	0.10	[−0.03, 0.23]	0.45
Leadership	**−0.22**	**[−0.34, −0.09]**	**32.28**	**0.28**	**[0.15, 0.40]**	**>100**	0.03	[−0.11, 0.16]	0.17
Forgiveness	−0.11	[−0.24, 0.02]	0.60	0.13	[0.00, 0.26]	0.99	0.05	[−0.08, 0.19]	0.21
Humility	**0.17**	**[0.04, 0.30]**	**3.79**	−0.10	[−0.23, 0.04]	0.42	0.11	[−0.02, 0.24]	0.61
Prudence	0.08	[−0.05, 0.21]	0.30	0.02	[−0.11, 0.15]	0.16	**0.20**	**[0.07, 0.33]**	**12.35**
Self–regulation	0.04	[−0.09, 0.17]	0.18	0.08	[−0.06, 0.21]	0.29	**0.30**	**[0.17, 0.41]**	**>100**
Appreciation	−0.04	[−0.17, 0.10]	0.18	0.15	[0.02, 0.28]	1.75	−0.11	[−0.24, 0.02]	0.57
Gratitude	−0.09	[−0.22, 0.05]	0.35	**0.19**	**[0.06, 0.32]**	**7.64**	0.07	[−0.07, 0.20]	0.25
Hope	**−0.17**	**[−0.30, −0.04]**	**3.93**	**0.36**	**[0.24, 0.47]**	**>100**	0.08	[−0.05, 0.21]	0.32
Humor	**−0.23**	**[−0.35, −0.10]**	**37.32**	**0.36**	**[0.24, 0.47]**	**>100**	**−0.17**	**[−0.30, −0.04]**	**3.94**
Spirituality	0.07	[−0.06, 0.20]	0.26	0.00	[−0.13, 0.14]	0.16	0.13	[0.00, 0.26]	0.96

*N* = 215–218; Appreciation = Appreciation of beauty and excellence; *CI* = confidence intervals; *BF* = Bayes Factor; bold = meaningful effects.

Predictability was negatively related to 12 of the 24 character strengths, with the largest negative correlations with creativity and kindness, and it showed a small positive correlation with humility. Overall, predictability showed a large overlap with the character strengths, *R*^2^ = 0.29, *p* < 0.001, *BF* > 100 (Beta weights for each character strength are displayed in Supplementary Table S6). Among the three flexibility aspects, adaptability showed the largest overlap with the character strengths: It was positively related to 17 of the 24 character strengths, with the largest relations to hope, humor, and bravery. The overlap between adaptability and character strengths was overall large, *R*^2^ = 0.35, *p* < 0.001, *BF* > 100. Orderliness showed the lowest overlap with the character strengths, and was positively related to six character strengths, with the largest correlations with perseverance and self-regulation, and it was negatively related to humor. Interestingly, the multiple correlation suggested a large overlap of orderliness with character strengths, *R*^2^ = 0.35, *p* < 0.001, *BF* > 100.

Overall, the three dimensions of flexibility showed different relationships with the 24 character strengths. As expected, the largest overlap with character strengths was found for adaptability, suggesting the virtuousness of this flexibility dimension. The large positive relationships with hope, humor, and bravery underline a common core of positive emotions and resilience shared between character strengths and adaptability. In line with our second prediction, predictability was mostly negatively related to the character strengths, with the largest negative correlations with creativity and kindness. Indeed, a person who likes things and circumstances to be predictable would not be motivated to look for new solutions for problems or challenges and novelty in everyday life. Regarding the third hypothesis, we found relatively few relationships between orderliness and character strengths. The largest correlations of orderliness were with perseverance and self-regulation, which suggests that the planning aspects of flexibility relate to continued and reliable work on tasks as well as controlling and managing oneself. At the same time, the regression indicated a large overlap of orderliness with character strengths. Hence, despite only a few stable effects, there were many small-sized or instable effects (both positive and negative ones) that accumulated when accounted together.

As character strengths are defined as positive personality qualities, it was the main aim of Study 1 to explore the relationships between the character strengths and flexibility on the level of typical behaviors. However, the study has some limitations. One limitation is the use of the initial version of the FS-24 to assess flexibility, rather than the final version (Vylobkova and Heintz, 2022a). One further limitation is the cross-sectional design of the study, which prevents any conclusions on the temporal relationships between flexibility and character strengths. In Study 2, these two limitations will be addressed. Further, we only used self-report measures. Future research should use other-reports, next to self-report measures, to validate the relationships between flexibility and the 24 character strengths, depending on the observability of these personality characteristics (for more details, see Vazire, 2010).

## 3. Study 2: Exploring the relationships between constructs in daily behaviors

Study 2 aimed to replicate and extend the results of Study 1. First, we sought to replicate the results in regard of the relationships between flexibility and the 24 character strengths at the level of typical behaviors. Second, we aimed to extend these relationships in a longitudinal design at the level of daily behaviors. For this purpose, we conducted a diary study with a pretest (typical behaviors) and a daily part over seven consecutive days (daily behaviors), to obtain specific behaviors that were shown on a given day.

According to the findings from Study 1, we hypothesize to replicate the correlation patterns between flexibility and character strengths for both typical and daily behaviors. The density distribution approach (Fleeson, 2001) from personality psychology postulates the similarity between typical behaviors and the average everyday behavior of the person. Further, Fleeson et al. (2002) suggests an isomorphism between the levels of typical and daily behaviors, which means that they share relevant properties. Furthermore, changes in daily behaviors seem to be one of the mechanisms for the change of a person’s typical behaviors (Hudson and Fraley, 2015). Therefore, the exploration of the relationships between flexibility and character strengths in daily life builds a solid basis for creating intervention programs aiming at fostering and changing daily flexibility and character strengths behaviors.

### 3.1. Materials and methods

#### 3.1.1. Procedure and participants

The data for Study 2 was collected as a part of a larger research project. The research project was preregistered prior to data collection.^1^ Parts of the data from the research project were used elsewhere (Vylobkova and Heintz, 2022a), though none of the research questions overlap with those of the current study. As in Study 1, participants were recruited online via social networks, personal contacts, and advertisements via mailing lists and in companies. Every participant provided informed consent before participation, and the study was conducted in accordance with the University’s ethics committee. The research project consisted of three parts: pretest, daily diary, and posttest. All questionnaires were completed online (via SosciSurvey.de). For Study 2, data from the pretest and daily diaries was used. The pretest consisted of demographic questions and of measures on typical behaviors for flexibility and character strengths. On the day after completing the pretest, participants received emails with links for the daily questionnaires. These emails were sent to participants in the evenings of the following 7 days. The daily surveys consisted of the character strengths measure followed by flexibility measures, and some questions about the day (i.e., on daily working hours, and further variables not related to the present study).

As an incentive, participants of the research project who completed the pretest and at least five daily measurements received individual feedback on their character strengths and work-life balance, and general recommendations for the strengths use at work and achieving greater work-life balance. An additional incentive for participants to complete all daily questionnaires was a donation of 1 Swiss franc for a social or environmental project chosen by the participant. The donation was made by the research team for every completed survey (pretest and all daily questionnaires).

The total sample size was defined according to the recommendations of Ohly et al. (2010) for daily studies in organizational research. A total of 195 participants completed the pretest. Five participants did not fulfill the inclusion criteria (workload ≥50%, age ≥ 18 years) and were excluded from analyses. As the estimated completion time for the pretest was 60 min, two participants were excluded due to a very short (less than 20 min) completion time. The final sample comprised 188 participants, whose mean age was 39.08 years (range 19–63), and 64.4% were women. The majority of the participants were Swiss (76.6%) and married or in a relationship (61.7%). The sample was rather well-educated with 72.3% holding a university degree or having a comparable education. Participants had an average workload of 83.5%.

#### 3.1.2. Instruments

In the pretest, similar to Study 1, the VIA-IS (Peterson et al., 2005; German version by Ruch et al. (2010) was used to assess the 24 character strengths. We again adopted the SAPA method (Revelle et al., 2017) to reduce the length of the survey for participants without impacting the reliability of the measures. Participants received 120 randomized VIA items instead of 240 (5 random blocks with 24 items each). To assess flexibility, the final version of the Flexibility Scale (FS-24; Vylobkova and Heintz, 2022a) was used. Supplementary Table S3 shows the reliabilities of the VIA-IS scales (range 0.60–0.91, median 0.73) and FS-24 (range 0.79–0.86, median 0.85).

To measure daily character strengths the *Character Strengths State Rating Form* (CSSRF; Gander et al., 2021) was used. We adapted the answer scale from a 7-point to 11-point scale from 0 (“never”) to 10 (“always”) in order to unify the answer scales of all daily items. To measure the daily expressions of flexibility dimensions, three self-constructed items (one item per flexibility dimension) were used. The items were constructed based on the item content of the FS-24 and are displayed in Supplementary Table S4.

#### 3.1.3. Statistical analyses

The data was analyzed with R software version 4.2.0. (R Development Core Team, 2020). We used the following R-packages: *BayesFactor* (Morey and Rouder, 2021), *dplyr* (Wickham et al., 2020), *Hmisc* (Harrell and Dupont, 2019), *psych* (Revelle, 2019), *lm.beta* (Behrendt, 2022), and *tidyverse* (Wickham et al., 2019). We first computed Pearson correlations between the 24 character strengths and flexibility on the level of typical behaviors (pretest). Further, we computed Tucker’s phi to compare the correlation patterns between flexibility and character strengths from Studies 1 and 2. In the third step, we computed the aggregated expressions of the character strengths and flexibility on the level of daily behaviors (daily diary) as well as Pearson correlations to explore the relationships between flexibility and character strengths on the daily level. Additionally, multiple correlation coefficients (regressions) with the 24 character strengths as predictors and flexibility aspects as criteria were calculated, separately for each level of behavior. We analyzed the effects analog to Study 1.

### 3.2. Results

#### 3.2.1. Level of typical behaviors

The descriptive statistics of the variables of Study 2 are given in Supplementary Table S3. The results of the Pearson correlations between the character strengths and flexibility on the level of typical behaviors are displayed in Table 2.

**Table 2:** Study 2: Pearson correlations between flexibility and VIA character strengths (pretest).

VIA character strengths	Flexibility subscales								
	Predictability			Adaptability			Orderliness		
	*r*	95% *CI*	*BF*	*r*	95% *CI*	*BF*	*r*	95% *CI*	*BF*
Creativity	**−0.32**	**[−0.44, −0.18]**	**>100**	**0.46**	**[0.34, 0.57]**	**>100**	**−0.23**	**[−0.36, −0.09]**	**27.10**
Curiosity	**−0.30**	**[−0.42, −0.16]**	**>100**	**0.45**	**[0.33, 0.56]**	**>100**	−0.09	[−0.23, 0.05]	0.35
Judgment	0.01	[−0.14, 0.15]	0.17	**0.20**	**[0.06, 0.33]**	**6.59**	0.13	[−0.01, 0.27]	0.78
Love of learning	**−0.23**	**[−0.36, −0.09]**	**24.00**	**0.34**	**[0.21, 0.46]**	**>100**	−0.05	[−0.20, 0.09]	0.22
Perspective	0.00	[−0.15, 0.14]	0.17	0.17	[0.03, 0.30]	2.30	0.13	[−0.01, 0.27]	0.87
Bravery	**−0.31**	**[−0.43, −0.17]**	**>100**	**0.42**	**[0.29, 0.53]**	**>100**	−0.12	[−0.26, 0.03]	0.60
Perseverance	−0.07	[−0.21, 0.07]	0.28	0.11	[−0.04, 0.25]	0.48	0.18	[0.03, 0.31]	2.86
Honesty	0.07	[−0.07, 0.21]	0.26	0.08	[−0.06, 0.22]	0.32	**0.19**	**[0.05, 0.32]**	**4.66**
Zest	**−0.21**	**[−0.34, −0.07]**	**9.42**	**0.26**	**[0.13, 0.39]**	**>100**	−0.01	[−0.15, 0.13]	0.17
Love	−0.04	[−0.19, 0.10]	0.20	0.16	[0.01, 0.29]	1.56	−0.05	[−0.19, 0.09]	0.21
Kindness	−0.11	[−0.25, 0.03]	0.52	0.14	[0.00, 0.28]	0.99	−0.04	[−0.18, 0.11]	0.19
Social intelligence	−0.02	[−0.17, 0.12]	0.18	0.16	[0.02, 0.30]	1.71	0.05	[−0.10, 0.19]	0.21
Teamwork	−0.08	[−0.22, 0.07]	0.29	0.10	[−0.04, 0.24]	0.43	0.07	[−0.08, 0.21]	0.25
Fairness	−0.17	[−0.31, −0.03]	2.52	**0.27**	**[0.13, 0.39]**	**>100**	−0.12	[−0.26, 0.02]	0.63
Leadership	−0.14	[−0.28, 0.00]	1.12	**0.26**	**[0.13, 0.39]**	**>100**	−0.06	[−0.20, 0.08]	0.23
Forgiveness	−0.12	[−0.26, 0.02]	0.66	0.17	[0.02, 0.30]	2.12	−0.07	[−0.21, 0.08]	0.26
Humility	0.15	[0.01, 0.29]	1.44	−0.05	[−0.19, 0.10]	0.21	0.13	[−0.02, 0.26]	0.70
Prudence	**0.23**	**[0.09, 0.36]**	**21.13**	0.00	[−0.15, 0.14]	0.17	**0.25**	**[0.11, 0.38]**	**59.59**
Self–regulation	0.07	[−0.08, 0.21]	0.27	0.08	[−0.06, 0.22]	0.30	**0.23**	**[0.09, 0.36]**	**20.63**
Appreciation	0.04	[−0.10, 0.18]	0.20	0.04	[−0.10, 0.18]	0.20	0.10	[−0.04, 0.24]	0.41
Gratitude	0.02	[−0.12, 0.16]	0.18	**0.22**	**[0.08, 0.35]**	**13.34**	0.16	[0.02, 0.29]	1.66
Hope	**−0.18**	**[−0.31, −0.04]**	**3.03**	**0.37**	**[0.24, 0.49]**	**>100**	−0.05	[−0.19, 0.09]	0.21
Humor	−0.17	[−0.31, −0.03]	2.41	**0.25**	**[0.11, 0.38]**	**58.45**	−0.13	[−0.27, 0.01]	0.87
Spirituality	0.08	[−0.07, 0.22]	0.28	0.04	[−0.10, 0.18]	0.19	0.11	[−0.03, 0.25]	0.55

*N* = 188; Appreciation = Appreciation of beauty and excellence; *CI* = confidence intervals; *BF* = Bayes Factor; bold = meaningful effects.

Predictability was negatively related to six character strengths, with the largest negative correlations found with creativity, curiosity, and bravery. It was also positively related to prudence. Adaptability again showed the largest overlap with character strengths, and was positively related to 11 character strengths. The largest correlations were found with creativity, curiosity, and bravery. Orderliness again showed the smallest overlap with character strengths, and was negatively related to creativity, and positively to honesty, prudence, and self-regulation. Furthermore, the results of the multiple correlations supported the varying relationships between the flexibility dimensions and character strengths, with the largest overlap for adaptability and the lowest for orderliness (*R_predictability_*^2^ = 0.38, *R_adaptability_*^2^ = 0.45, *R_orderliness_*^2^ = 0.33; all *p*s < 0.001, all *BF*s > 100). (Beta weights for each character strengths are displayed in Supplementary Table S6).

To compare the similarity of the relationships between flexibility and character strengths on the level of typical behaviors obtained in Studies 1 and 2, the Tucker’s phi congruency coefficients was computed. Tucker’s phi across the 72 correlations (3 flexibility factors × 24 character strengths) was 0.85, suggesting a fair similarity of the correlation patterns between the two studies (Lorenzo-Seva and ten Berge, 2006).

#### 3.2.2. Level of daily behaviors

The convergence between typical and daily behavior levels for each construct can be found in Supplementary Table S5. All convergence levels were sufficiently high and comparable to previous research findings (Wagner and Gander, 2022). This indicates that our measurement of typical and daily behaviors converged and can be meaningfully interpreted (see Supplementary Table S5).

**Table 3:** Study 2: Pearson correlations between flexibility and VIA character strengths (daily questionnaires).

VIA character strengths	Flexibility subscales								
	Predictability			Adaptability			Orderliness		
	*r*	*95% CI*	*BF*	*r*	*95% CI*	*BF*	*r*	*95% CI*	*BF*
Creativity	0.11	[−0.08, 0.30]	0.42	**0.61**	**[0.48, 0.72]**	**>100**	0.20	[0.00, 0.37]	1.49
Curiosity	0.13	[−0.07, 0.31]	0.50	**0.59**	**[0.45, 0.70]**	**>100**	0.14	[−0.05, 0.33]	0.62
Judgment	0.14	[−0.06, 0.32]	0.56	**0.51**	**[0.35, 0.64]**	**>100**	0.15	[−0.05, 0.33]	0.65
Love of learning	0.19	[0.00, 0.37]	1.32	**0.51**	**[0.35, 0.64]**	**>100**	0.22	[0.03, 0.40]	2.80
Perspective	0.21	[0.02, 0.39]	2.13	**0.51**	**[0.36, 0.64]**	**>100**	**0.29**	**[0.10, 0.45]**	**14.15**
Bravery	**0.24**	**[0.05, 0.41]**	**4.14**	**0.45**	**[0.29, 0.59]**	**>100**	**0.29**	**[0.10, 0.45]**	**15.44**
Perseverance	**0.27**	**[0.08, 0.44]**	**8.76**	**0.39**	**[0.22, 0.54]**	**>100**	**0.32**	**[0.14, 0.49]**	**51.37**
Honesty	0.17	[−0.02, 0.35]	0.91	**0.49**	**[0.32, 0.62]**	**>100**	0.18	[−0.02, 0.36]	1.03
Zest	0.15	[−0.04, 0.33]	0.68	**0.46**	**[0.30, 0.60]**	**>100**	0.17	[−0.02, 0.35]	1.00
Love	0.20	[0.01, 0.37]	1.53	**0.33**	**[0.15, 0.49]**	**62.84**	**0.30**	**[0.11, 0.46]**	**20.82**
Kindness	0.17	[−0.02, 0.35]	0.98	**0.51**	**[0.35, 0.64]**	**>100**	**0.25**	**[0.06, 0.42]**	**4.87**
Social intelligence	0.10	[−0.10, 0.28]	0.36	**0.58**	**[0.43, 0.69]**	**>100**	0.19	[0.00, 0.37]	1.42
Teamwork	**0.26**	**[0.08, 0.43]**	**7.41**	**0.49**	**[0.33, 0.62]**	**>100**	**0.26**	**[0.08, 0.43]**	**7.37**
Fairness	0.20	[0.00, 0.37]	1.50	**0.59**	**[0.45, 0.71]**	**>100**	**0.26**	**[0.07, 0.43]**	**6.46**
Leadership	0.21	[0.02, 0.38]	1.90	**0.51**	**[0.35, 0.64]**	**>100**	**0.25**	**[0.07, 0.42]**	**5.74**
Forgiveness	**0.33**	**[0.15, 0.49]**	**72.43**	**0.53**	**[0.38, 0.66]**	**>100**	**0.45**	**[0.28, 0.59]**	**>100**
Humility	**0.36**	**[0.18, 0.52]**	**>100**	**0.39**	**[0.21, 0.54]**	**>100**	**0.38**	**[0.20, 0.53]**	**>100**
Prudence	**0.40**	**[0.22, 0.55]**	**>100**	**0.46**	**[0.29, 0.60]**	**>100**	**0.47**	**[0.30, 0.60]**	**>100**
Self–regulation	**0.36**	**[0.19, 0.52]**	**>100**	**0.40**	**[0.22, 0.55]**	**>100**	**0.39**	**[0.21, 0.54]**	**>100**
Appreciation	**0.36**	**[0.18, 0.52]**	**>100**	**0.39**	**[0.21, 0.54]**	**>100**	**0.44**	**[0.28, 0.59]**	**>100**
Gratitude	**0.37**	**[0.20, 0.53]**	**>100**	**0.36**	**[0.18, 0.52]**	**>100**	**0.34**	**[0.16, 0.50]**	**96.36**
Hope	0.21	[0.02, 0.39]	2.03	**0.49**	**[0.33, 0.62]**	**>100**	**0.30**	**[0.11, 0.46]**	**19.89**
Humor	−0.01	[−0.20, 0.18]	0.23	**0.39**	**[0.22, 0.54]**	**>100**	−0.04	[−0.23, 0.15]	0.24
Spirituality	**0.23**	**[0.04, 0.40]**	**3.12**	**0.25**	**[0.06, 0.42]**	**5.29**	**0.24**	**[0.04, 0.41]**	**3.50**

*N* = 104–105; Appreciation = Appreciation of beauty and excellence; *CI* = confidence intervals; *BF* = Bayes Factor; bold = meaningful effects.

In contrast to the typical behaviors, only positive correlations between flexibility and character strengths as daily behaviors were found. Adaptability was related positively to all character strengths, with the largest correlations for creativity, curiosity, fairness, and social intelligence. Predictability was positively related to 10 character strengths, with the largest correlations with prudence and gratitude. Orderliness was positively related to 16 character strengths, with the largest correlations for prudence, forgiveness, and appreciation of beauty and excellence. The overall overlaps with the character strengths for each flexibility aspect were again large, with the largest effect for adaptability (*R_predictability_*^2^ = 0.39, *p* < 0.05, *BF* = 0.41; *R_adaptability_*^2^ = 0.59, *p* < 0.001, *BF* > 100; *R_orderliness_*^2^ = 0.47, *p* < 0.001, *BF* = 29, beta weight for each character strength are displayed in the Supplementary Table S6), though the Bayes Factor for predictability remained inconclusive.

To further compare the results of the typical and daily behaviors from Study 2, we computed Tucker’s phi. The congruency coefficients of the correlations was 1, suggesting equality of the correlation patterns between the two levels of analysis (Lorenzo-Seva and ten Berge, 2006). Thus, although the correlations were overall more positive and stronger in the daily, compared of the typical behaviors, the general pattern (i.e., largest and smallest correlations between flexibility factors and character strengths) can be considered equal.

### 3.3. Discussion

Study 2 aimed at replicating the results of the typical behaviors, and extending them to daily behaviors. The results from Study 1 were replicated, with the largest overlap between flexibility and 24 character strengths found for the adaptability dimension. Predictability was mostly negatively correlated, and orderliness was the least related to the 24 character strengths. Tucker’s phi suggested that the correlation patterns for the typical behavior measures across the two studies were stable. This further supports the notion that flexibility can overall be seen as a positive and morally valued personality characteristic, and that considering the three factors offers nuance in interpreting the concept within positive psychology. Specifically, adaptability seems the most positive and morally valued, and predictability the least.

Regarding the relationships between character strengths and flexibility at the level of daily behaviors, we found overall a larger overlap between the constructs compared to the level of typical behaviors. The adaptability dimension showed the most consistent positive relationships with character strengths. Predictability was positively related to the character strengths at the daily level, in contrast to the mostly negative relationships at the level of typical behaviors. Consistent positive relationships emerged between predictability and prudence across both levels of behavior, indicating commonality in careful and planning behaviors. The orderliness dimension showed a larger overlap with character strengths at the daily level in comparison to the level of typical behaviors. Consistent positive relationships emerged between orderliness and prudence as well as self-regulation across both levels of behavior. Hence, acting orderly was related to being more modest and self-controlled.

While the direction and size of the correlations differed between daily and typical behavior, Tucker’s phi showed that the pattern of correlations (i.e., the rank order of character strengths that are more or less related to the flexibility dimensions) was equal. This supports the theoretical notions of the density distribution approach and isomorphism between the levels of typical and daily behaviors (Fleeson, 2001; Fleeson et al., 2002) for the first time for the relationships between flexibility and character strengths. As a consequence, findings using typical behaviors can potentially be generalized to daily behaviors, opening exciting possibilities for flexibility interventions as avenues in positive-psychological research (e.g., Seligman et al., 2005; Gander et al., 2013), as has already been done in psychotherapies, such as Acceptance and Commitment Therapy (e.g., Wersebe et al., 2018; Puolakanaho et al., 2020).

Analog to Study 1, this study is limited due to self-report measures. Further, for exploring the construct-related daily behaviors we used accumulated values, which do not allow to account for within-person variance. Although this was sufficient for the purpose of this study, future research could adopt different analysis strategies that allow to examine the within-person variance and the dynamics of flexibility and character strengths in daily behaviors (Fleeson and Noftle, 2012).

## 4. General discussion

This two-part research aimed to explore the relationships between flexibility and the 24 character strengths of the CSV classification (Peterson and Seligman, 2004). As character strengths are seen as positive, moral and virtuous personal qualities, an empirical investigation of these relationships allows us to infer how positive, virtuous and moral flexibility is as a personal characteristic. For this purpose, we explored the relationships between flexibility and character strengths on the level of typical behaviors in two studies. In Study 2, we also expanded the scope by exploring the relationships between constructs on the level of daily behaviors.

The aim of Study 1 was the initial exploration of the relationships between flexibility and the 24 character strengths at the level of typical behaviors. The largest overlap with character strengths was found for the adaptability dimension. Predictability was mostly negatively related to the 24 character strengths, and orderliness showed the fewest relationships with character strengths. These initial results suggest that especially the adaptability dimension can be seen as positive, moral and virtuous, as it showed the largest overlap with the character strengths. These findings were corroborated in Study 2, with Tucker’s phi indicating a fair similarity between these correlations. This suggests that positive-psychological interventions would best focus on the adaptability dimension of flexibility to maximize the positive effect. It also shows the importance of distinguishing these three flexibility dimensions in positive-psychological research and applications, as just using an overall flexibility scale might mix positive and moral aspects with more neutral or even negative and immoral components, confounding the potential relevance of the flexibility dimensions for positive traits and experiences. This also suggests that the malleability of the three flexibility dimensions in longitudinal studies and interventions should be studied separately, as some dimensions might be more stable than others, in addition to being related to different outcomes.

Further, Study 2 expanded the results of Study 1 by investigating the relationships between flexibility and character strengths on the level of daily behaviors. At the daily level, adaptability was related positively to all 24 character strengths, predictability was related positively to 10, and orderliness to 16 character strengths. The results suggest that in daily life, not only being able to adapt, but also being able to plan things in advance and to define the areas of performance were positive and closely related to daily manifestations of character strengths. In other words, at the daily level, a larger overlap between flexibility and character strengths was found in comparison to the level of typical behaviors. Despite these apparent differences, Tucker’s phi showed that the pattern of correlations was equal, supporting Fleeson’s density distribution approach (Fleeson, 2001; Fleeson et al., 2002). This further supports the notion that flexibility behaves similar to other more established personality characteristics (e.g., extraversion; Fleeson, 2001; Fleeson et al., 2002), and that studies and interventions that target daily flexibility behaviors will likely yield similar patterns to habitual behaviors. For instance, general relationships of flexibility and the three dimensions to well-being can potentially be generalized to daily manifestations of flexibility, which can in turn be targeted using positive-psychological interventions, analog to character-strengths based interventions (e.g., Seligman et al., 2005; Gander et al., 2013).

Overall, the results from the two studies suggested the virtuousness of the adaptability dimension at the level of typical and daily behaviors, as this flexibility dimension showed the largest overlap with the 24 character strengths across the two studies. Predictability and orderliness instead seemed to be positively related to the character strengths in daily life rather than at the level of typical behaviors, where mostly negative (predictability) or inconsistent overlap (orderliness) with character strengths were found. One possible explanation here could be a challenging time during which the data of the Study 2 was collected (June to December 2020), which was marked by bringing almost every day new information and rules for the society. Therefore, some predictability and ability to plan everyday life was possibly related to increased control over one’s life circumstances and was perceived as positive during the challenging times (e.g., Glynn et al., 2021). This raises the potential idea that each flexibility dimension could be beneficial given the circumstances the individual is in; for example, being orderly and creating some predictable circumstances might allow an individual to behave in this “safe space” adaptively. The interplay between the three dimensions in relation to other typical and daily behaviors, as well as well-being outcomes, is an important avenue for future research. This can include additive (each dimension independently), synergistic (dimensions reinforcing each other), or compensatory (high values in one dimension compensating for low values in others) effects, determining the best combination of the three flexibility dimensions (see Trautwein et al., 2015, for an investigation of these effects in the personality context).

Regarding specific character strengths, adaptability showed a consistent positive relationship with 11 strengths (creativity, curiosity, judgment, love of learning, bravery, honesty, fairness, leadership, gratitude, hope, and humor). The largest overlap was found with creativity, curiosity, bravery, and hope over the two levels of analysis and two studies. Indeed, to be able to adapt own thoughts, plans, and behaviors to new situations needs creativity (for creating novel ideas and ways of actions), curiosity (for exploring further possible options in a new situation), bravery (for facing novel situations and challenges), and hope (belief that everything will turn out well). These findings are in line with previous research on the overlap between openness to experiences, creativity, and curiosity (Dametto and Noronha, 2021) as well as adaptability (Vylobkova and Heintz, 2022a).

For predictability, the most consistent relationships over the two studies (in 2 of the 3 analyses) were found with humility and prudence (positive), and with hope, zest, bravery, love of learning, curiosity, and creativity (negative). The largest effects were identified for prudence, humility and creativity. It seems that people who value certain and predictable environments tend to plan carefully, are modest, but at the same time do not engage as much in divergent thinking (finding many different solutions to problems), which is in line with previous findings on the need for cognitive closure and a preference for convergent problem-solving tasks (i.e., one solution to a problem; Wronska et al., 2019).

For orderliness, the most consistent relationships (at least 2 of 3 analyses) were found with perseverance, prudence and self-regulation (positive) and with creativity (negative). Indeed, to pursue a goal, a structured plan is needed that is pursued in the long run, as well as the ability to regulate and control own thoughts and emotions. Similar to predictability, being orderly relates to more convergent, conventional thinking. These results are also in line with previous findings on orderliness and conscientiousness (Vylobkova and Heintz, 2022a).

### 4.1. Limitations and directions for future research

When interpreting the findings of the present research, some methodological aspects need to be considered. First of all, both studies use self-report measures. Although self-reports are a common practice in psychology, future research could adopt other-reports to study the expressions of the flexibility and character strengths on the level of typical behaviors, and in daily behaviors (see Vazire, 2010).

Further, the sample size of the studies needs to be taken into account. Although we assume the stability of correlations at a sample size between 150 and 250 (Schönbrodt and Perugini, 2013), and followed recommendations on sample sizes by Ohly et al. (2010), the reliability of the instruments needs to be considered (Kretzschmar and Gignac, 2019), which can greatly increase the demand for sample sizes. Therefore, the replication of the present results in a larger sample would be desirable. Although the convergence between assessment instruments for character strengths is high (Vylobkova et al., 2022), replications of the current results using other instruments to assess the 24 CSV strengths would be of interest,. Finally, the design of the studies has several limitations. Study 1 adopted cross-sectional and Study 2 cross-sectional and longitudinal designs. A period of 7 days for a daily diary could be somewhat short for the observation of the entire spectrum of variability in daily behaviors. Although we followed Ohly et al.’s (2010) suggestion that the course of 5 days is a minimum for a diary study, Fleeson et al. (2002) suggested a study over 9–15 days to observe the entire range of the expressions related to the typical behaviors in everyday life. Future studies could adopt longer-term longitudinal designs, as well as intensive longitudinal designs with several data collections each day (see Bolger and Laurenceau, 2013), to help to gain more fine-grained insights into the dynamics of the relationships between the studied constructs. Furthermore, future research could additionally adopt an experimental design to be able to make conclusions on the causal relationships between flexibility and character strengths; this could be done by adapting character-strengths or flexibility-based positive psychology interventions (Schutte and Malouff, 2019; Lavy, 2020).

### 4.2. Theoretical and practical implications

The current research is the first to explore the positive and moral side of flexibility. The fact that flexibility is one of the 21^st^ century competences (Ananiadou and Claro, 2009) indicates that it is seen as a positive and desirable personal characteristic. In the present research, we explored a further indicator of the positivity of flexibility, namely its relationships with positive personal qualities (24 character strengths). The results support the theoretical assumption of Lavy (2020) that flexibility cannot be transferred to a single character strength, and that it is rather closely related to a number of character strengths. Recent studies underlined the importance of flexibility and related concepts for well-being (e.g., Doorley et al., 2020). The current research is in line with these studies and provides further support of the positivity and morality of flexibility as a personality characteristic. Future research can expand this area of investigation, for example by studying the role of flexibility for specific aspects of well-being or its role for specific life domains, such as the workplace, schools, or romantic relationships.

Especially in the context of positive psychology, the current results can have broad implications for research and practice. As one of the aims of positive psychology is to study the positive sides of human nature (Seligman and Csikszentmihalyi, 2000), a number of positive personality aspects are in the focus, such as resilience (Smith et al., 2010), psychological capital (Luthans et al., 2007), mindfulness (Lomas et al., 2019), and character strengths (McGrath, 2015). The current results expand these positive personality aspects to include flexibility. Therefore, it would be interesting to investigate the relationships between flexibility and further well-studied positive aspects of human nature and to integrate flexibility in positive-psychological research. Furthermore, the role of flexibility for desired outcomes and different aspects of well-being needs to be investigated further. As a positive and desirable aspect of personality, frameworks and tools for fostering flexibility are needed and build a promising branch for future research. This can draw from established positive psychology interventions, such as those based on character strengths (Schutte and Malouff, 2019; Lavy, 2020), as well as previous applications within psychotherapies (e.g., Wersebe et al., 2018; Puolakanaho et al., 2020) Additionally, the present research contributes to a better understanding of the relationships between character strengths and other positive traits, which has recently been extended to resilience (Martínez-Martí and Ruch, 2017) and environmental self-efficacy (Moeller and Stahlmann, 2019).

The current research suggested that flexibility is a positive and moral characteristic of a person and is closely related to the character strengths. Therefore, considering or including flexibility in existing positive-psychological and strengths-based interventions could positively influence the effects of the given intervention. As most research on flexibility and related concepts comes from work and organizational psychology, an extension of existing interventions to the workplace seems both feasible and desirable. For instance, employees who used their own character strengths in a new way more often had a greater sense of calling towards their work (Harzer and Ruch, 2016). The extension of this intervention by flexibility exercises could have the purpose to make participants more flexible and open to new and unconventional ways of using their strengths at work. This could enhance the effectiveness of the intervention on calling. In general, the well-studied intervention on using own strengths in new ways (Seligman et al., 2005) could be supported by flexible behaviors in applying the new ways to strengths use. Also, existing interventions could target some forms of flexibility in the workplace context, such as for burnout (Puolakanaho et al., 2020), performance (Bray et al., 2018), and job crafting (Wessels et al., 2020; Tims et al., 2022).

Speaking more generally, for any psychological intervention, a flexibility boost prior to intervention activities could be helpful. This could make the participants more open to the intervention activities and to the changes induced (e.g., daily writing of three positive things that happened during the day; Seligman et al., 2005). Indeed, extraversion and openness to experiences as personality qualities were found to enhance the positive effects of positive-psychological interventions on well-being (Senf and Liau, 2013). As these personality characteristics were strongly correlated with flexibility (Vylobkova and Heintz, 2022a), flexibility exercises might have a similar effect on enhancing their effectiveness.

### 4.3. Conclusion

Flexibility as a positive aspect of human nature is closely and strongly related to the 24 character strengths. As the first study on the relationships between the 24 character strengths and flexibility, it builds a solid basis for integrating flexibility into the positive psychological literature. The results of the present and future research would eventually allow to design intervention programs aimed at developing and fostering positive aspects of human nature, relevant in the modern, challenging and changing world.

## Data availability statement

The datasets presented in this article are not readily available because due to ethical reasons (participants were not asked about their agreement to share the data publicly), supporting data is not available. Requests to access the datasets should be directed to VV, v.vylobkova@psychologie.uzh.ch.

## Ethics statement

Ethical approval was not provided for this study on human participants because in accordance with the ethical requirements of the institution no ethical approval was needed for this study. The patients/participants provided their written informed consent to participate in this study.

## Author contributions

VV: conceptualization, formal analysis, investigation, writing–original draft and writing–review and editing. SH: conceptualization and writing–review and editing. All authors contributed to the article and approved the submitted version.

## Funding

This publication was supported by the Swiss National Science Foundation (grant number 172′723).

## Conflict of interest

The authors declare that the research was conducted in the absence of any commercial or financial relationships that could be construed as a potential conflict of interest.

## Publisher’s note

All claims expressed in this article are solely those of the authors and do not necessarily represent those of their affiliated organizations, or those of the publisher, the editors and the reviewers. Any product that may be evaluated in this article, or claim that may be made by its manufacturer, is not guaranteed or endorsed by the publisher.

## Footnotes

^1^ https://aspredicted.org/blind.php?x=cs85rh
