# Supplementary material for: A meeting of positive behaviors: The relations of three aspects of flexibility with character strengths
Source: Front Psychol. 2023 Feb 3;13:1078764. doi: 10.3389/fpsyg.2022.1078764 (PMC9935571; doi:10.3389/fpsyg.2022.1078764)
Supplement: Supplementary file 1 [file Data_Sheet_1.docx]

Supplementary materials

Table S1

Character strengths and virtues classification (CSV, Peterson & Seligman, 2004)

| Core virtues | Character strengths |
| --- | --- |
| Wisdom and knowledge | Creativity |
|  | Curiosity |
|  | Judgment |
|  | Love of learning |
|  | Perspective |
| Courage | Bravery |
|  | Perseverance |
|  | Honesty |
|  | Zest |
| Humanity | Love |
|  | Kindness |
|  | Social intelligence |
| Justice | Teamwork |
|  | Fairness |
|  | Leadership |
| Temperance | Forgiveness |
|  | Humility |
|  | Prudence |
|  | Self-regulation |
| Transcendence | Appreciation of beauty and excellence |
|  | Gratitude |
|  | Hope |
|  | Humor |
|  | Spirituality |

Table S2

Descriptive Statistics of the Study 1 variables

| Scales | *M* | *SD* | *Sk* | *K* |
| --- | --- | --- | --- | --- |
| VIA strengths |  |  |  |  |
| Creativity | 3.53 | 0.74 | -0.43 | 0.06 |
| Curiosity | 3.92 | 0.64 | -0.33 | -0.42 |
| Judgment | 3.89 | 0.56 | -0.29 | 0.08 |
| Love of learning | 3.82 | 0.71 | -0.64 | 0.91 |
| Perspective | 3.59 | 0.58 | -0.18 | 0.01 |
| Bravery | 3.60 | 0.62 | -0.05 | -0.35 |
| Perseverance | 3.58 | 0.66 | -0.67 | 0.35 |
| Honesty | 3.89 | 0.51 | -0.08 | -0.16 |
| Zest | 3.59 | 0.62 | -0.33 | -0.25 |
| Love | 3.73 | 0.68 | -0.25 | -0.39 |
| Kindness | 3.83 | 0.56 | -0.15 | 0.07 |
| Social intelligence | 3.69 | 0.62 | -0.20 | -0.04 |
| Teamwork | 3.67 | 0.59 | -0.52 | 1.57 |
| Fairness | 3.93 | 0.57 | -0.67 | 0.85 |
| Leadership | 3.64 | 0.54 | -0.16 | -0.08 |
| Forgiveness | 3.54 | 0.67 | -0.58 | 0.87 |
| Humility | 3.32 | 0.68 | -0.25 | 0.23 |
| Prudence | 3.34 | 0.60 | 0.14 | -0.03 |
| Self–regulation | 3.24 | 0.68 | -0.23 | -0.53 |
| Appreciation | 3.53 | 0.72 | -0.26 | -0.19 |
| Gratitude | 3.82 | 0.68 | -0.33 | -0.28 |
| Hope | 3.58 | 0.59 | -0.38 | 0.42 |
| Humor | 3.71 | 0.67 | -0.13 | -0.43 |
| Spirituality | 2.80 | 0.97 | 0.34 | -0.50 |
| FS-24 |  |  |  |  |
| Predictability | 3.04 | 0.87 | 0.06 | -0.80 |
| Adaptability | 4.11 | 0.59 | -0.56 | 0.44 |
| Orderliness | 3.30 | 0.78 | -0.22 | -0.41 |

*Note*. Appreciation – Appreciation of beauty and excellence, FS-24 – initial version of the FS-24, *Sk* – Skewness, *K* - Kurtosis

Table S3

Descriptive Statistics of the Study 2 variables

|  | *Pretest* | | | | | *Daily questionnaires* | | | | |
| --- | --- | --- | --- | --- | --- | --- | --- | --- | --- | --- |
| Scales | *M* | *SD* | *Sk* | *K* | α | *M* | *SD* | *Sk* | *K* | α |
| VIA strengths |  |  |  |  |  |  |  |  |  |  |
| Creativity | 3.57 | 0.77 | -0.57 | -0.12 | .89 | 6.72 | 1.67 | -0.18 | 0.01 | .82 |
| Curiosity | 4.01 | 0.59 | -0.54 | 0.08 | .79 | 7.38 | 1.49 | -0.23 | -0.09 | .77 |
| Judgment | 3.83 | 0.57 | -0.32 | -0.07 | .84 | 7.44 | 1.40 | -0.50 | 0.85 | .77 |
| Love of learning | 3.73 | 0.70 | -0.28 | -0.72 | .83 | 6.76 | 1.70 | -0.04 | 0.03 | .79 |
| Perspective | 3.57 | 0.51 | -0.61 | 0.64 | .70 | 7.06 | 1.43 | -0.44 | -0.17 | .78 |
| Bravery | 3.61 | 0.59 | -0.20 | 0.30 | .78 | 6.25 | 1.69 | -0.07 | -0.70 | .75 |
| Perseverance | 3.55 | 0.61 | -0.23 | 0.03 | .84 | 7.65 | 1.40 | -0.50 | -0.37 | .66 |
| Honesty | 3.81 | 0.48 | -0.25 | -0.30 | .66 | 8.49 | 1.31 | -0.57 | 0.86 | .82 |
| Zest | 3.71 | 0.59 | -0.21 | -0.36 | .73 | 7.43 | 1.48 | -0.33 | -0.08 | .73 |
| Love | 3.90 | 0.57 | -0.45 | 0.14 | .71 | 7.94 | 1.55 | -1.01 | 1.93 | .84 |
| Kindness | 3.79 | 0.53 | 0.00 | -0.42 | .62 | 7.95 | 1.33 | -0.47 | 0.20 | .77 |
| Social intelligence | 3.77 | 0.50 | -0.48 | 0.13 | .72 | 7.92 | 1.33 | -0.75 | 0.48 | .81 |
| Teamwork | 3.73 | 0.52 | -0.36 | 0.34 | .73 | 6.77 | 1.91 | -0.75 | 0.11 | .81 |
| Fairness | 4.02 | 0.46 | -0.23 | -0.08 | .65 | 7.43 | 1.50 | -0.51 | 0.53 | .84 |
| Leadership | 3.72 | 0.53 | -0.13 | -0.46 | .66 | 6.08 | 2.06 | -0.50 | -0.52 | .79 |
| Forgiveness | 3.55 | 0.54 | -0.04 | 0.17 | .69 | 5.88 | 1.97 | -0.22 | -0.68 | .86 |
| Humility | 3.21 | 0.62 | -0.27 | -0.23 | .73 | 6.65 | 1.75 | -0.29 | -0.46 | .88 |
| Prudence | 3.28 | 0.54 | -0.10 | -0.47 | .60 | 7.46 | 1.45 | -0.32 | -0.14 | .81 |
| Self–regulation | 3.25 | 0.63 | -0.05 | -0.01 | .69 | 7.44 | 1.46 | 0.00 | -0.26 | .78 |
| Appreciation | 3.59 | 0.65 | -0.01 | -0.84 | .74 | 6.87 | 1.67 | -0.01 | -0.17 | .79 |
| Gratitude | 3.86 | 0.56 | -0.38 | -0.29 | .74 | 7.30 | 1.85 | -0.58 | -0.33 | .87 |
| Hope | 3.65 | 0.59 | -0.53 | 0.12 | .76 | 7.50 | 1.44 | -0.28 | 0.00 | .78 |
| Humor | 3.73 | 0.62 | -0.73 | 0.78 | .82 | 6.98 | 1.73 | -0.56 | 0.42 | .86 |
| Spirituality | 2.94 | 1.01 | 0.23 | -0.84 | .91 | 4.01 | 2.90 | 0.79 | -0.50 | .97 |
| FS-24 |  |  |  |  |  |  |  |  |  |  |
| Predictability | 3.69 | 1.03 | -0.22 | -0.61 | .86 | 6.16 | 1.92 | -0.21 | -0.81 | .92 |
| Adaptability | 5.26 | 0.79 | -0.51 | 0.07 | .79 | 7.94 | 1.33 | -0.43 | -0.35 | .81 |
| Orderliness | 4.63 | 1.04 | -0.40 | -0.63 | .85 | 6.77 | 2.10 | -0.20 | -0.83 | .93 |

*Note*. Appreciation – Appreciation of beauty and excellence, FS-24 – final version of the FS-24, α = Cronbach`s α.

Table S4

Flexibility items for daily questionnaires

| Flexibility dimension | Items English | Items German |
| --- | --- | --- |
| Predictability | People who value predictability like to plan the course of their day precisely and prefer to do activities where they can estimate what to expect. They feel most comfortable when everything is going their usual way or when their activities are confined to a clearly defined area. They perform better when their environment remains stable. They often find changes in their lives threatening and frustrating. They are reluctant to engage in activities that require them to act quickly. | Vorhersehbarkeit - Personen, die Vorhersehbarkeit schätzen, mögen es, den Ablauf ihres Tages genau zu planen und erledigen lieber Tätigkeiten, bei denen sie abschätzen können, was sie erwartet. Sie fühlen sich am wohlsten, wenn alles seinen gewohnten Gang hat oder wenn ihre Tätigkeiten auf einen klar definierten Bereich beschränkt bleiben. Sie erbringen bessere Leistungen, wenn ihr Umfeld stabil bleibt. Veränderungen in ihrem Leben empfinden sie oft als bedrohlich und frustrierend. Sie beschäftigen sich ungern mit Tätigkeiten, die ein schnelles Handeln verlangen. |
| Adaptability | People who are adaptable can quickly adjust themselves, their own behavior and plans to changing and unexpected situations. They cope well with changing conditions and perform well in ambiguous situations. From time to time they abandon the familiar and do something completely new. For them, things are not just "black or white". | Anpassungsfähigkeit - Personen, die anpassungsfähig sind, können sich, eigenes Verhalten und Pläne schnell an wechselnde und unerwartete Situationen anpassen. Sie können gut mit sich verändernden Bedingungen umgehen und erbringen gute Leistungen in unklaren Situationen. Von Zeit zu Zeit geben sie das Gewohnte auf und machen etwas völlig Neues. Für sie sind die Dinge nicht nur "schwarz oder weiss". |
| Orderliness | People who appreciate order and rules like to plan things in advance and proceed according to the German proverb "order is half of life". They make sure that everything they do is well planned and everything has its fixed place. They do things in a proven way if possible and don't like disorder. | Ordnungsliebe - Personen, die Ordnung und Regeln schätzen, planen Dinge gerne im Voraus und verfahren nach dem Sprichwort "Ordnung ist das halbe Leben". Sie achten darauf, dass alles, was sie tun, gut geplant ist und jedes Ding seinen festen Platz hat. Sie machen Dinge möglichst auf bewährte Art und Weise und mögen keine Unordnung. |

*Note*. Instruction for daily Flexibility questionnaire was: “Below you see a series of statements about habits and behaviors that may apply to you. Please indicate on a scale from "never" to "always" how **often** you have exhibited these habits and behaviors **today**.” The answer scale is from 0 (never) to 10 (always).

Table S5

Convergence between typical and daily behaviors for character strengths and flexibility

| Scale | Convergence |
| --- | --- |
| Character strengths |  |
| Creativity | .50 |
| Curiosity | .33 |
| Judgment | .43 |
| Love of learning | .35 |
| Perspective | .26 |
| Bravery | .31 |
| Perseverance | .26 |
| Honesty | .32 |
| Zest | .33 |
| Love | .43 |
| Kindness | .33 |
| Social intelligence | .27 |
| Teamwork | .23 |
| Fairness | .33 |
| Leadership | .37 |
| Forgiveness | .36 |
| Humility | .44 |
| Prudence | .33 |
| Self-regulation | .33 |
| Appreciation of beauty and excellence | .36 |
| Gratitude | .48 |
| Hope | .47 |
| Humor | .49 |
| Spirituality | .75 |
| Flexibility dimensions |  |
| Predictability | .53 |
| Adaptability | .43 |
| Orderliness | .75 |

*Note*. N = 104-105

Table S6

Betas of the multiple regression analyses for predicting flexibility dimensions in Study 1 and Study 2

| VIA character strengths | Study one | | | Study two | | | Study two | | |
| --- | --- | --- | --- | --- | --- | --- | --- | --- | --- |
|  |  |  |  | Traits | | | States | | |
|  | Predictability | Adaptability | Orderliness | Predictability | Adaptability | Orderliness | Predictability | Adaptability | Orderliness |
| Creativity | -.10 | .11 | -.14 | -.16 | .29 | -.18 | -.05 | .19 | .06 |
| Curiosity | -.01 | .17 | -.07 | -.19 | .22 | -.11 | .06 | .13 | -.16 |
| Judgment | .02 | .08 | .09 | .10 | .05 | .17 | -.44 | .00 | -.41 |
| Love of learning | -.12 | .01 | -.10 | -.17 | .14 | -.07 | -.12 | .09 | -.14 |
| Perspective | -.11 | .06 | -.06 | .04 | -.10 | .07 | .23 | -.07 | .26 |
| Bravery | -.07 | .04 | .11 | -.19 | .22 | -.14 | .00 | -.05 | .03 |
| Perseverance | .11 | -.03 | .12 | -.07 | -.05 | .13 | .15 | -.14 | .19 |
| Honesty | .10 | .01 | .18 | .18 | -.06 | .20 | .21 | .03 | .02 |
| Zest | -.11 | -.03 | .25 | -.09 | -.13 | -.01 | -.05 | .25 | -.03 |
| Love | -.09 | -.04 | -.09 | .07 | .01 | -.07 | .02 | -.13 | .30 |
| Kindness | -.25 | .16 | -.09 | -.08 | -.11 | -.06 | -.21 | .13 | -.15 |
| Social Intelligence | -.02 | .14 | -.01 | .06 | .01 | .05 | -.30 | .25 | -.28 |
| Teamwork | -.11 | .10 | -.13 | -.08 | -.01 | .09 | .33 | .09 | .19 |
| Fairness | .05 | -.03 | .05 | -.12 | .08 | -.18 | -.23 | .15 | -.19 |
| Leadership | -.13 | .07 | .00 | -.08 | .05 | -.18 | -.03 | .07 | .02 |
| Forgiveness | -.03 | .04 | .00 | -.04 | .00 | -.05 | .22 | .14 | .31 |
| Humility | .09 | -.06 | -.05 | .06 | .05 | .07 | .09 | .00 | -.01 |
| Prudence | .16 | -.21 | .19 | .27 | -.14 | .10 | .22 | .01 | .41 |
| Self–regulation | .11 | .04 | .17 | .05 | .07 | .11 | .36 | -.12 | .22 |
| Appreciation | .14 | -.03 | -.17 | .17 | -.22 | .11 | .14 | -.01 | .15 |
| Gratitude | .08 | -.03 | .11 | .11 | .08 | .22 | .21 | -.28 | -.07 |
| Hope | -.11 | .20 | -.09 | .07 | .16 | .02 | -.21 | -.03 | .01 |
| Humor | .02 | .08 | -.17 | -.08 | .12 | -.07 | -.12 | .13 | -.15 |
| Spirituality | .10 | -.08 | .14 | .04 | .05 | .07 | -.13 | .17 | -.13 |
| R^2^ | .29 | .35 | .35 | .38 | .45 | .33 | .39 | .59 | .47 |

*Note*. Appreciation – Appreciation of beauty and intelligence. *N* = 104-188.
